# What explains low adoption of digital payment technologies? Evidence from small-scale merchants in Jaipur, India

**DOI:** 10.1371/journal.pone.0219450

**Published:** 2019-07-31

**Authors:** Ethan Ligon, Badal Malick, Ketki Sheth, Carly Trachtman

**Affiliations:** 1 Department of Agricultural and Resource Economics, University of California, Berkeley, Berkeley, California, United States of America; 2 CATALYST, IFMR LEAD, New Delhi, India; 3 Department of Economics, University of California, Merced, Merced, California, United States of America; Shandong University of Science and Technology, CHINA

## Abstract

The availability of digital payment technologies (such as internet banking, mobile money, and credit/debit cards) has rapidly increased in the developing world, and is a cornerstone for financial inclusion initiatives in developing countries. Despite significant efforts to promote digital payments, rates of adoption remain modest in some low-income countries. In particular, the rate of adoption in India remains low despite significant efforts to promote adoption. In this paper, we consider possible reasons for the low rates of adoption among merchants in Jaipur, India with small fixed-location store enterprises. Using survey data for 1,003 merchants, we find little evidence that supply-side barriers to obtaining necessary infrastructure or meeting prerequisite requirements to adopt digital payments explain the low level of adoption. Merchants are able to obtain infrastructure to transact digitally (such as bank accounts and smart phones), fees on digital platforms are affordable, and merchants are sufficiently literate to be able to use digital payment systems. We conclude that adoption is both feasible and inexpensive. Therefore, low rates of adoption do not appear to be the result of supply-side barriers, but due rather to demand-side factors or taxes. We find direct evidence of such demand-side factors, such as a perceived lack of customers wanting to pay digitally, and concerns that records of mobile payments might increase tax liability. Our results thus suggest that simply lowering the costs associated with adopting these technologies is unlikely to be successful in increasing adoption of digital payments.

## Introduction

In recent years, digital transaction methods have become a cornerstone for financial inclusion policies. Mobile money platforms have been introduced in more than 90 countries [1], and 30–40 billion dollars per year are devoted to digital inclusion initiatives by large international funders [2]. There are several possible benefits of digital payments: reducing frictions of transacting in cash (e.g., making change), reducing distance-related costs (e.g., depositing cash), increasing financial transparency (e.g., cash-related fraud), increasing security (e.g., reducing theft of cash), and improving business record-keeping (creating a verifiable history of transactions, which can help facilitate interaction with the formal financial sector) [3] [4]. Increased transparency may also provide economy-wide benefits, such as improved tax compliance. Given these potential benefits, in some contexts like East Africa, digital transactions have become commonplace. For instance, the mobile money platform M-PESA in Kenya has reached almost universal coverage of the adult population [5] and has led to a 2% reduction in the overall number of poor households [6]. However, the growth of digital payments is uneven and some places continue to have very low rates of adoption. While in 2016 China had over 4 credit/debit cards and approximately 0.2 Point of Sale (PoS) terminals (to accept card payments) per capita, India had less than one card and only about 0.002 point of sale terminals per capita, for instance [7].

The case of India is particularly interesting; India’s economy remains mostly cash-based despite the existence of various types of digital payments. The Government of India has recently made substantial efforts to reduce the use of cash. Some notable efforts include: the November 2016 demonetization of the 500 and 1,000 Rupee bank notes [8]; the creation of India Stack (a collection of APIs which leverage the Aadhaar biometrically-verified identification program to make digital transactions cheaper and more efficient) [9]; and a large-scale incentive program between April 2017 and July 2018 (in which the Government of India offered small monetary incentives to merchants for accepting payments through a government-supported digital payment system called Unified Payments Interface or UPI from their customers, and to consumers for referring new UPI users) [10].

Despite these efforts, digital payment adoption in India remains relatively low among firms and consumers alike, and cash still prevails; the cash-to-GDP ratio was already back to pre-demonetization levels by May 2018 [11]. Given the supposed benefits of digital payments, a natural question arises: why has there has not been a more widespread shift to digital payment usage in India? Though the behavior of both consumers and firms is important to understand, here we focus on the digital payment adoption decisions of firms, specifically merchants that sell products to consumers. A potential explanation for low adoption among firms is that there are “supply-side” barriers that serve as binding constraints, inhibiting adoption of digital payments. We consider supply-side barriers here as essentially costs (set by suppliers of digital payments systems and suppliers of related infrastructure) that inhibit a firm from adopting digital payments (i.e. firms would choose to adopt if prices were lower). More specifically, in order to use digital payment technologies, firms must possess the necessary physical infrastructure (e.g., smart phones), have access to the internet, have a mobile-linked bank account, be able to pay the usage fees associated with the technology, and have sufficient literacy/technological literacy to use the product. Without satisfying these prerequisites, it may be prohibitively expensive for firms to adopt such technologies, as they would have to shoulder the costs associated with satisfying them, such as buying a smart phone. Generally, such supply-side factors to adopting technologies that seem profitable (at least when used in developing countries) are a common explanation in the development economics literature for low technology adoption, which is why, for example, many studies in the literature focus on credit constraints as a hindrance to adoption of a profitable technology (as firms would like to adopt a technology that is profitable, but they cannot afford to buy without credit, which they have difficulty obtaining) [12] [13]. Hence motivated by this literature, we consider the effect of supply-side barriers on adoption as a starting point to guide our analysis.

### Objectives and framework

To understand merchants’ adoption decisions we adopt a simple microeconomic framework. This framework treats adoption as a fundamentally economic decision, driven by merchants’ assessments of the salient costs and benefits. Examples of costs merchants might consider might include on-going payments made to internet providers or to payment service providers, or the fixed costs of purchasing hardware such as smartphones or point of service (PoS) terminals. Examples of benefits might include simpler and more transparent bookkeeping; increased demand (for the merchants goods or services) from consumers who prefer to use mobile payment technologies rather than cash; or reduced risks of theft and reduced costs of transacting with banks. At the broad industry level, we will describe the costs merchants consider to be “supply-side” factors, since the costs small merchants face will generally depend on the behavior and technology used by firms building mobile money platforms and providing services to the small merchants we consider. Obversely, we’ll think of the benefits considered by merchants to be “demand-side” factors, since these will typically depend on the needs and resources available to the merchants’ customers.

Within our microeconomic framework, our chief objective is to test the hypothesis that supply-side (rather than demand side) factors are the principal obstacle to digital payments adoption by merchants in Jaipur. This is not the only hypothesis one could test, but is of particular policy interest because of a focus by the government of India and international organizations on reducing these costs as a means toward spurring adoption.

Our simple microeconomic framing of the problem is not the only possible framing of the decision merchants face, and other frameworks might be more suitable for testing other hypotheses. For example, a more behavioral approach called the “Technology Adoption Model” (TAM) with origins in social psychology focuses less on cost-benefit calculations and more on on intrinsic motivations and would-be users’ perceptions of the usefulness and ease-of-use of a new technology [14]. Related approaches in the marketing literature [15] also pay close attention to the details of customers’ perceptions of new technologies and their resulting behavioral reactions. These approaches would be better suited than ours to measuring variation in adoption related to variations in technology, such as different software applications having different user interfaces. In contrast, a more “macro” approach models the spread of a new technology as a diffusion process [16]. Such an aggregate approach would be better suited to understanding things such as the relationship between aggregate economic growth and technological innovation.

Our microeconomic approach requires neither the data on perceptions of usefulness and ease of use employed in the TAM approach nor the international data on national income and product accounts exploited in the macro literature, and focuses instead on answering a particular question: do the costs borne by would-be merchant adopters deter adoption of digital payments technology?

### Preview of results

Typical of the low adoption observed in India, we find that about 60% of merchants in our sample have not adopted digital payments of any type. Our survey data suggests that the previously described supply-side barriers do not fully explain the low rates of digital payments adoption in our sample. We demonstrate this is in three distinct ways. First, we show that 98.6% of sample firms are feasible prospective digital payment users, in that they have the necessary documents, business income, and literacy so that they *could* satisfy all of the prerequisites for digital payment adoption if they so chose (i.e. they could afford to purchase necessary infrastructure, have the documents to open a bank account, etc.). Second, we show that not only are there many merchants that could satisfy these prerequisites, but many merchants *already do* satisfy them. In our sample, 97% of merchants have a bank account, 79% have a device with the potential to access the internet if purchased (such as a smart phone or computer), 55% have internet access, just under 100% can afford the related usage fees, and 96% are technologically literate. In total, 54.24% of the sample merchants already satisfy all of the aforementioned requirements, and yet only 42.0% of the sample have adopted digital payments. Third, we show that even among current digital payment users, usage is low, with around 80% of their transactions with customers still being done in cash. Given these three findings, we conclude that lack of digital payment adoption by sample merchants is generally not due to being unable to overcome supply-side barriers for using digital transaction methods. Instead, decisions not to adopt may depend mainly on perceptions of other demand-side benefits or taxes (which, from the merchants’ point of view have the same effect on profits as a reduction in demand). We present evidence (including regression analysis) that beliefs about customer demand to pay digitally, and beliefs about increased tax liabilities associated with digital payments (from the merchants’ point of view an increased tax has the same effect as a reduction in consumer demand) may play important roles in merchants’ decisions to adopt digital payments.

The rest of the paper proceeds as follows. The “Materials and methods” section presents additional background assumptions used in our estimation about the types of digital payment technologies available to merchants in the sample and the necessary infrastructure required to adopt these technologies, a description of the data, and a description of analytical methods used. The “Results” section lists our main results suggesting supply-side barriers are not the main cause of non-adoption of digital payment technologies by merchants in our sample. The “Discussion” section provides alternate explanations of digital payment non-adoption based on demand-side factors, and discusses determinants of digital payment adoption. Our final thoughts are contained in the “Conclusion” section.

## Materials and methods

The Institutional Review Board at the University of California, Berkeley, has granted a formal waiver of ethical approval (Protocol ID:2017-03-9720) for this research, which relies on survey data we obtained from PRICE (http://www.ice360.in/). Survey participants provided informed written consent to have data from the survey used in research; all survey data used in this paper were fully anonymized before being accessed by us for analysis.

We explore the role of supply-side factors in merchants’ adoption decisions by analyzing survey data from 1,003 small-scale fixed store merchants in Jaipur, Rajasthan collected in 2017. In this section we first provide definitions and background assumptions to establish context, then discuss the data, including sampling procedures, aspects of the survey, and descriptive statistics. This is followed by a discussion of our methods.

### Key definitions and background assumptions

#### Small-scale fixed store merchants

“Fixed store merchants” are defined here as businesses conducting enterprise activities that occur within fixed premises/permanent structures outside the household, generally in market locations. This category does not include street vendors, home-based business, and service providers, all of which are also common in Jaipur [17]. A “small-scale” enterprise is defined according to criteria established by the Indian government, and requires that total investment be less than fifty million INR for manufacturers, or less than twenty million INR for enterprises in the service sector. Small-scale fixed store merchants are of specific interest because they tend to be formalized enough to have links with banks and strong growth potential, but may still have trouble accessing credit through the formal financial sector. Additionally, the recent implementation of a Goods and Services Tax (GST), creates incentives for careful record-keeping among taxpaying firms. Adoption of digital payment technologies addresses both of these issues, creating a formalized transaction history which can aid in verification of transactions completed and taxes paid.

#### Types of digital payments

There were four main types of relevant digital payment technologies for merchants in Jaipur at the time of the survey. The first is internet banking platforms, which allow transactions between individuals’ bank accounts to be completed through a bank website or app from a computer or mobile phone, often with associated transaction fees. This is essentially the digital equivalent of exchanging a check. The second type is PoS devices, which allow merchants to accept credit/debit card payments, usually through a physical card swipe. The third type is mobile wallets, in which participants move money to a digital “wallet” within a mobile app and can transfer money to others’ in-app wallets (manually or by scanning a QR code). However, one potential barrier to transacting using this method is that many companies offer mobile wallets to consumers and merchants in India, and it generally is not possible to transfer money from one company’s mobile wallet to another’s without an intermediate transfer. The fourth digital payment type is the UPI, a system developed and run by the not-for-profit National Payments Corporation of India (NPCI) and overseen by the Reserve Bank of India, which allows individuals to transfer money to each other directly between bank accounts though mobile apps, such as Bharat Interface for Money (BHIM) and QR code scanning. Unlike online banking platforms, UPI immediately transfers money whenever a payment is made, including on weekends and holidays, and unlike mobile wallets, any two people with bank accounts can transfer money. However, UPI is also much newer than the other digital payment types; it was founded in April 2016, which was much later than many popular mobile wallets were established (two top mobile wallets in India, PayTM and Mobikwik, were founded in 2010 and 2009 respectively).

#### Prerequisites for digital payment adoption

In order for merchants to accept digital payments, there are prerequisites they must satisfy; namely merchants need to have a bank account, have an internet connection, have a device with internet access, be able to pay the fees associated with using these devices, and be technologically literate enough to use digital payments. If merchants do not already satisfy these prerequisites, they may face supply-side barriers to obtaining documents and/or paying costs associated with adoption. Assumptions used in this analysis regarding prerequisites to use digital payments for one year and how to satisfy them are summarized in Table 1.

**Table 1:** Prerequisites necessary for adopting digital payment technologies for one year.

Prerequisite	Required documents	Estimated cost	Other
Bank account	Aadhaar and PAN		
Device (Smartphone)	Aadhaar (or other ID proof)	INR 3,228 (US$48.42)	
Internet (and phone service)		INR 3,614 (US$54.21)	
Usage fees (Mobile Wallet)		INR 2,400 (US$36.00)	
Technological literacy			Written literacy
**All**	Aadhaar and PAN	INR 9,245 (US$138.63)	Written literacy

Note: Document requirements and cost estimates for bank accounts, devices, and internet are based on informal interviews and online investigation carried out by the authors. Estimated usage fees are based on survey data information provided by merchants. All costs are estimated at the one year level, given that we only have profit estimates at the year level. US Dollar amounts are listed in parenthesis using a conversion rate of INR 1 = US$ 0.015.

All four digital payment types require merchants to have bank accounts at public or private banks in order to complete transactions, either because transactions are done directly between consumers’ and merchants’ bank accounts (for internet banking and UPI) or because they are done using card or wallet platforms in which money must be transferred in and out from a bank account (for PoS devices and mobile wallets). If merchants do not satisfy the bank account prerequisite already, they will be able to open an account fairly easily as long as they have the required documents. At the time of the survey, these were an Aadhaar card and a Permanent Account Number (PAN) card [18] [19], which are documents used to verify citizens’ identity (Aadhaar) and taxpayer status (PAN).

The “device” in Table 1 refers to a device through which the merchant can access the website or application necessary to make and accept digital payments. All four digital payment types can be completed over a smartphone application, hence when we estimate costs of obtaining a device, we do so for the case of a smartphone. However, internet banking can be done using a laptop or desktop computer, and some mobile wallet payments can be done using an internet-capable feature phone [20]. Therefore when we consider whether merchants already have the appropriate device to accept digital payments, we suppose this is the case if they have a smartphone, feature phone with internet capabilities, or computer. We estimate the costs of buying a smartphone that would work well with digital payments at INR 7,000 (US$105.00) [21]. Additionally, to use a smartphone one must also obtain a SIM card, for which we estimate a cost of INR 100 (US$1.50) and which requires a proof of identity document (such as Aadhaar) to obtain [22]. Hence in total, the costs are INR 7,100. Yet, we are calculating annual costs and a new smartphone is not purchased every year. In many emerging markets, smartphones are replaced every 2.2 to 2.5 years [23]. Hence we divide these costs by 2.2, to calculate an annual device cost of about INR 3,228 (US$48.42).

Aside from the cost of owning a phone, there are costs for the phone to be functional, including the costs of cell service and internet data (which are also necessary to transact digitally). Cell service and data are extremely inexpensive in India; an investigation on the World Bank Data Blog found that in absolute terms, India was sixth cheapest country in the world in terms of monthly mobile phone fees in 2014, at approximately US$2.80 [24], and Cable.co.uk found that in 2018, India was the country with the cheapest price of cellular data, an average price of of US$0.26/GB [25]. To estimate costs of purchasing cell phone service and data, we consider more current information about smartphone plans for one of India’s biggest telecom providers, Airtel [26]. An example of a current pre-paid “bundled” 10-day plan offered by Airtel in Rajasthan is unlimited calls to and from most places in India, 1 GB of 3G/4G data, and 100 text messages within India a day all for 99 Indian Rupees (INR). This plan is fairly comprehensive, would likely meet a typical merchant’s needs, and would only cost INR 3,614 for an entire year (US$54.20); for our calculations we round this up to INR 3,700.

Some digital payment systems have additional upfront costs (such as the cost of purchasing a PoS device to swipe cards), as well as recurring service fees (either a flat fee per month or a fee per transaction). In our survey data (explained in detail in the following section), merchants who use digital payments were asked about the upfront and recurring costs associated with various digital payment methods. The median reported value for each can be seen in Table 2. We note that while accepting internet banking and PoS payments do seem to be associated with significant upfront fees, mobile wallets and UPI are not. Instead, mobile wallets have some small recurring fees associated with them, though these recurring fees tend to only be the fees associated with transferring (a large enough sum of) money out of the mobile wallet and into a bank account, and hence may not be incurred every month [27]. For the estimated costs in this paper, we suppose the fees associated with using digital payments for a year are INR 2,400. We chose this number by calculating the median cost of using any digital payment for a year (multiplying the monthly fee by 12 and adding this to the upfront cost) among sample merchants.

**Table 2:** Median digital payment usage fees.

Type	*N*	Upfront fee	Monthly fee
Internet banking	114	1, 250	300
PoS	127	2, 500	300
Mobile wallet	346	0	120
UPI	15	0	0

Note: All fee-related statistics in the table are medians, given outliers in the right tail of the distributions (suggesting that some merchants may have factored in other infrastructure costs not directly related to digital payments). Costs were only reported by merchants that use any given digital payment method; these numbers of merchants reporting are listed under *N*. Monthly fees include the sum of any recurring costs that accrue within a month.

Finally, to use digital payments, merchants must either have an underlying general knowledge of how to use digital technology or be literate enough to be able to figure out how to do so. While we do not have exact information on whether a merchant is “literate” enough to use a smartphone, we can look at proxies, such as general literacy, education level, current smartphone app use, current digital payment technology use, and whether they report one of their employees being comfortable with a smartphone (as even if the owner is not technologically literate, an employee may be able to complete transactions). For the purposes of our analysis, we consider a merchant technologically literate enough if they have completed at least eighth grade in school, currently use digital payments, have used social media smartphone apps, and/or have at least one employee who can comfortably use a smartphone, and consider a merchant to have baseline “written literacy” such that they could potentially obtain technological literacy, if they do not report being illiterate.

### Data

The survey data in this study was collected as part of a project headed by Catalyst, an initiative funded by United States Agency for International Development (USAID) under the mSTAR Program [28], to increase adoption of digital payments in India, using a targeted ecosystem approach. The survey interviewed 1,003 fixed store merchants in Jaipur, and was conducted by People Research on India’s Consumer Economy (PRICE) in August-September 2017, following a listing census that identified 6,011 households and enterprises in Jaipur a few months prior in April–May 2017. More information about the sampling strategy, which was random, but with stratification by industry and “digital readiness,” can be found in the S1 Appendix. The goal of the survey was to learn more about the ecosystem in which merchants interact in Jaipur, and specifically to understand the adoption of and willingness to use digital payment technologies by merchants. Jaipur is a tier two city in India, whose citizens span the socioeconomic distribution and may be representative of other medium size urban areas in India (some other tier two cities include Agra, Lucknow, Chandigarh, and Nagpur). Jaipur was selected as a “lab” because Jaipur is a focus area for Catalyst, and the Government of Rajasthan is interested in increasing digital payment adoption among citizens. But more than that, Jaipur is an interesting context to study; as a medium-sized urban area with large socioeconomic diversity, and many small and medium-sized merchants, it is a setting where many merchants could potentially gain from adopting digital payments. The survey collected various information, including personal information about the business owners’ households, information about the business operations and profits, and attitudes toward digital payment technologies. Additionally, when available, statistics from the listing census will also be presented. All data used in this paper can be found at https://osf.io/qzj2t/. More information about sample merchants can also be found in [29].

Sample businesses operate within a variety of different industries, with the most common being apparels, fabrics, and furnishing, general stores, and food service activities (which together comprise over 40% of all sample businesses). Almost all sample businesses are permanent, perennial, sole proprietorships. Over two-thirds of businesses were started by their current owners, and the average business has been operating under its current owner for 16.5 years. Additionally, these businesses are relatively formal; not only do they have a permanent structure, but over four-fifths report being registered with the Ministry of Corporate Affairs. Despite being relatively well-established, most businesses report that they want to grow. The majority are retail stores doing most of their sales with consumers as opposed to other businesses. The sample does contain some wholesale stores and service providers as well. Generally, these stores are well-trafficked, receiving a median of 95 customer visits weekly, with about 90 visits ending in a transaction. Businesses receive many repeat customers; on average businesses report that 40% of all customers are repeat customers. Generally, the size of these businesses is small to medium-sized, with a median of two employees per firm, aside from the owner. About half of these employees are hired labor and the other half are unpaid family labor. Average annual sales are INR 2,147,522 (US$32,212), and average annual profits are INR 381,441 (US$6,000).

The survey also collects information on the owners of these businesses. In terms of demographics, almost all of the merchants in our sample are male. The majority are Hindu, the majority religion in the area, and most of the rest are Muslim. About a third are a member of a Scheduled Caste, a Scheduled Tribe, or an Other Backward Class. Nearly all of the merchants in the sample have an Aadhaar identification number that will allow them to access financial services such as a bank account. Additionally, these merchants are quite educated; almost eighty percent have at least a tenth grade education. Only ten merchants in the entire sample (less than 1%) report that they are completely illiterate. In terms of these merchants’ households, the average merchant has about 6.25 household members. In general, most of these households’ income comes from their business; on average 85%. Per capita income is a little less than US$1,500 annually, and per capita expenditures is closer to US$730. These numbers suggest that in terms of socio-economic status, these households are similarly well-off to other urban areas in India, and close to the average national GNI per capita, which was approximately US$1,820 in 2017 [30].

### Methods

We use descriptive methods supported by linear regression analysis to show that supply-side costs cannot plausibly account for low rates of digital payments adoption. To do this, we characterize sample businesses as digital payment users if they report they are currently using one of the four digital payment technologies listed above in their business. We compare the percentage of digital payment user businesses to the percentage of businesses that could feasibly satisfy all of the prerequisites to adopt digital payments. A business is considered a feasible digital payment user if for each of the five prerequisites listed in Table 1 they meet one of the following criteria:

1. The business already satisfies that prerequisite (e.g. the business already has a smart phone).
2. The business has the necessary documents, annual profits (which they could use to purchase devices/cover fees), or literacy to satisfy the prerequisite if they so chose. (e.g. the business does not have a smart phone, but their profits exceed INR 3,228 a year, suggesting it is feasible for the business to obtain a functional smart phone using their profits).

The idea behind this comparison exercise is that if satisfying these prerequisites is the primary barrier to digital payment adoption, then the number of businesses that are able to satisfy the prerequisites should be similar to the number of businesses that accept digital payments. This follows from the reasoning that those who do not adopt are unable to because they cannot satisfy the prerequisites. If instead many more merchants can feasibly satisfy the prerequisites but are still not adopting digital payments, this implies that though merchants can theoretically adopt digital payments, they do not find accepting digital payments to be an optimal use of their resources; in other words, that the perceived costs of adoption do not outweigh the perceived benefits.

There may be a concern that our cost estimates do not accurately capture the full costs of satisfying the prerequisites to adopt digital payments, and hence that some “feasible” users may face significant barriers to adopting digital payments. To address this, we compare the percentages of businesses using digital payments to the percentage of businesses that already satisfy the requirements for digital payments(i.e. they have a bank account, device, and internet service, can afford service fees, and appear technologically literate). If these prerequisites are the binding constraints to adoption, we would expect these percentages to be similar. If instead more merchants satisfy the prerequisites but are not adopting digital payments, this again implies that some other features in the digital payment technologies are generally driving non-adoption.

Finally, these previous exercises considered adoption as a binary choice, that is the decision of the business is whether to use any digital payments at all. Yet anecdotal evidence suggests that many merchants have “adopted” digital payments in the sense that they could accept these, but in practice seldom do. Hence, we also explore digital payment usage among the users. If barriers to satisfying prerequisites for digital payment adoption are the main binding constraint to adoption, then we would expect that those businesses that do use digital payments (and hence have overcome any relevant supply-side barriers) use them quite liberally. If this is not the case, this would support the conclusion that there are other important factors or reasons that drive businesses to not adopt digital payments in this context.

## Results

In this section, we first present evidence of non-adoption of digital payments among sample merchants, showing that 58% of sample merchants have not adopted digital payments of any type. Then we present evidence that prerequisites that may act as barriers (having to open a bank account, obtain a smart phone, obtain internet access, pay usage fees, and be literate) are not the primary reasons for non-adoption among the sample. We do this in three parts. First, we show that 98.6% of sample firms are feasible prospective digital payment users, in that they already satisfy the prerequisites for digital payment adoption or they have the necessary documents, business income, and baseline literacy such that they could realistically choose to satisfy all of the prerequisites. Second, we show that not only are there many sample merchants that *could* satisfy these prerequisites, but 54.2% merchants *already* satisfy them. Third, we show that even among current digital payment users, usage is low, with 81.4% of their transactions with customers still being done in cash.

### Digital payment adoption

A summary of digital payment use in the sample, including a breakdown of the type of digital payment methods used, can be seen in Table 3. In both the listing census and final survey, about 40% of fixed store merchants (36.3% in the listing and 42.0% in the main survey) already use a digital payment technology of some type. The most common type of technology used is mobile wallets, with 82.1% of all digital payment users in the main sample being mobile wallet users. After that, 30.2% of all digital users in the main survey sample use PoS devices, slightly less use internet banking, and very few use UPI. Again, despite the fact that UPI seems to be a dominant technology to the other methods, it was a fairly new technology at the time, likely explaining these low numbers.

**Table 3:** Digital payment users.

Type	% Businesses (Census)	% Businesses (Main Survey)
Internet banking	10.5%	11.4%
PoS	13.5%	12.7%
Mobile wallet	28.6%	34.5
UPI	3.1%	1.5%
**Any of the above**	36.3%	42.0%

Note: In both the census and main survey, merchants were asked which digital mediums they/their enterprise uses for payments out of internet banking, PoS/mPoS devices, mobile wallets, and UPI. Additionally, the final row presents the percentage of merchants that reported using any of the four preceding technologies (hence it is the union of the previous measures). One merchant with missing census data was excluded from this census data statistics.

### Feasibility of digital payment adoption

Now we consider the question whether merchants are unable to adopt digital payment technologies because they are in some sense unable to satisfy the requirements necessary to adopt digital payments. The main results can be seen in Table 4.

**Table 4:** Ability of sample businesses to adopt digital payments based on ability to satisfy prerequisites.

Requirement	Have means to obtain	Already have	Have means to obtain or already have
Bank account	92.2%	96.6%	99.3%
Smart phone/Device	99.7%	79.3%	100%
Internet access	99.7%	55.5%	99.8%
Usage fees	99.7%	99.7%	99.7%
Technological literacy	99.0%	96.3%	99.6%
**All combined**	91.5%	54.2%	98.6%

Note that in the case of “Usage fees” the second and third columns are the same by construction, since one cannot “have” usage fees, but rather have sufficient profits with which to pay the fees. The final “All combined” row details the percentage of merchants satisfying all the above criteria for each column (for the first column: they have Aadhaar and PAN cards, at least INR 9,245 in profits, and baseline literacy, for the second column: they have a bank account, a device, internet access, technological literacy as defined in the previous section, and profits exceeding INR 2,400, for the third column: they either already have or have means to access all of the requirements combined).

The second column of Table 4, labeled “Have means to obtain,” reports the percentage of merchants that have the resources (in terms of documents, profits to cover costs, and baseline literacy) to obtain each requirement, as detailed in Table 1. To understand whether merchants can cover costs for a given requirement, we consider whether their annual profits exceed the annual costs associated with the requirement. For the vast majority of sample merchants, having enough annual profits to cover the costs associated with digital payment use does not seem to be an issue, with over 99% of merchants being able to cover all of the associated fees. Additionally, 99% of sample merchants have at least basic literacy, such that with some effort, they could learn how to navigate a smart phone application to accept digital payments. We observe fewer merchants satisfying the requirements for having a bank account. While over 99% of merchants have the required Aadhaar card to open a bank account, only about 93% have a PAN card. While there are costs associated with not having a PAN card (major financial transactions are impossible to complete legally), there are also significant costs associated with getting a PAN card if a merchants does not have one (namely, they likely have to start paying taxes). Hence for the 7% of merchants without a PAN card, there may be significant barriers to opening a bank account. Thus, we find that 91.53% of merchants are feasibly able to obtain all of the requirements to use digital payments.

We further observe that some merchants satisfy the requirements (Column 2 of Table 4), despite not meeting the technical requirements of doing so (Column 1 of Table 4). Notably, we see that while only 92% of the merchants technically have the means to get a bank account, almost 97% report actually having a bank account. This discrepancy could potentially be explained in a variety of ways; perhaps the bank account a merchant uses is actually under some other person’s name, or perhaps the merchant was able to open an account previously (given the changing nature of recent laws surrounding the documents required). To ensure we include merchants that already have the required infrastructure, such as a bank account, we calculate the final column labeled “Have means to obtain or Already have”. This is the union of qualifying merchants in the previous two columns, yielding the percentage of all the merchants that either could obtain or already have each piece of infrastructure. We are left with the striking result that 98.6% of sample businesses are able to satisfy the prerequisites associated with digital payment use. This is significantly greater than the 40% of merchants that actually use digital payments in our sample, and so is evidence that the ability to satisfy these prerequisites is not the binding constraint inhibiting digital payment adoption. Additionally, this suggests by revealed preference that merchants do not see digital payments as providing an annual benefit that exceeds the annual costs of INR 9,245.

There may be concerns that though merchants’ profits exceed the costs of obtaining the requirement, that these costs may comprise a significant portion of business profits, and that business profits are a major part of their household income that cannot easily be spared. In this vein, there are a few important points to note. First, for the median sample merchant, all of the annual costs combined are only 3% of their annual profits. Additionally, if we think that businesses only adopt if digital payment-related costs make up a small enough fraction of their profits, we note that these costs only comprise more than 10% of annual profits for 5.28% of the sample and comprise more than 5% of profits for 25.1% of the sample. Moreover, out of those merchants for which annual costs would theoretically comprise more than 5% of their profits, 35.7% have already adopted digital payments, which is broadly similar to adoption in the full sample. To see this relationship visually in S1 Fig, we plot the empirical cumulative distribution function of annual profits and indicate the fraction of merchants whose profits fall below each of these thresholds. Additionally, to the point that business profits cannot be spared because they are essential to household expenditure, the average differential between household income and expenditures is INR 247,800, which is almost 27 times more than all of the prerequisite costs combined. Additionally, only 44 sample merchants (4.39% of the sample) report a difference between income and expenditures that is less than INR 9,245. Out of these 44 merchants, 34% use digital payments already.

### Possession of the underlying requirements

**Fig 1:**
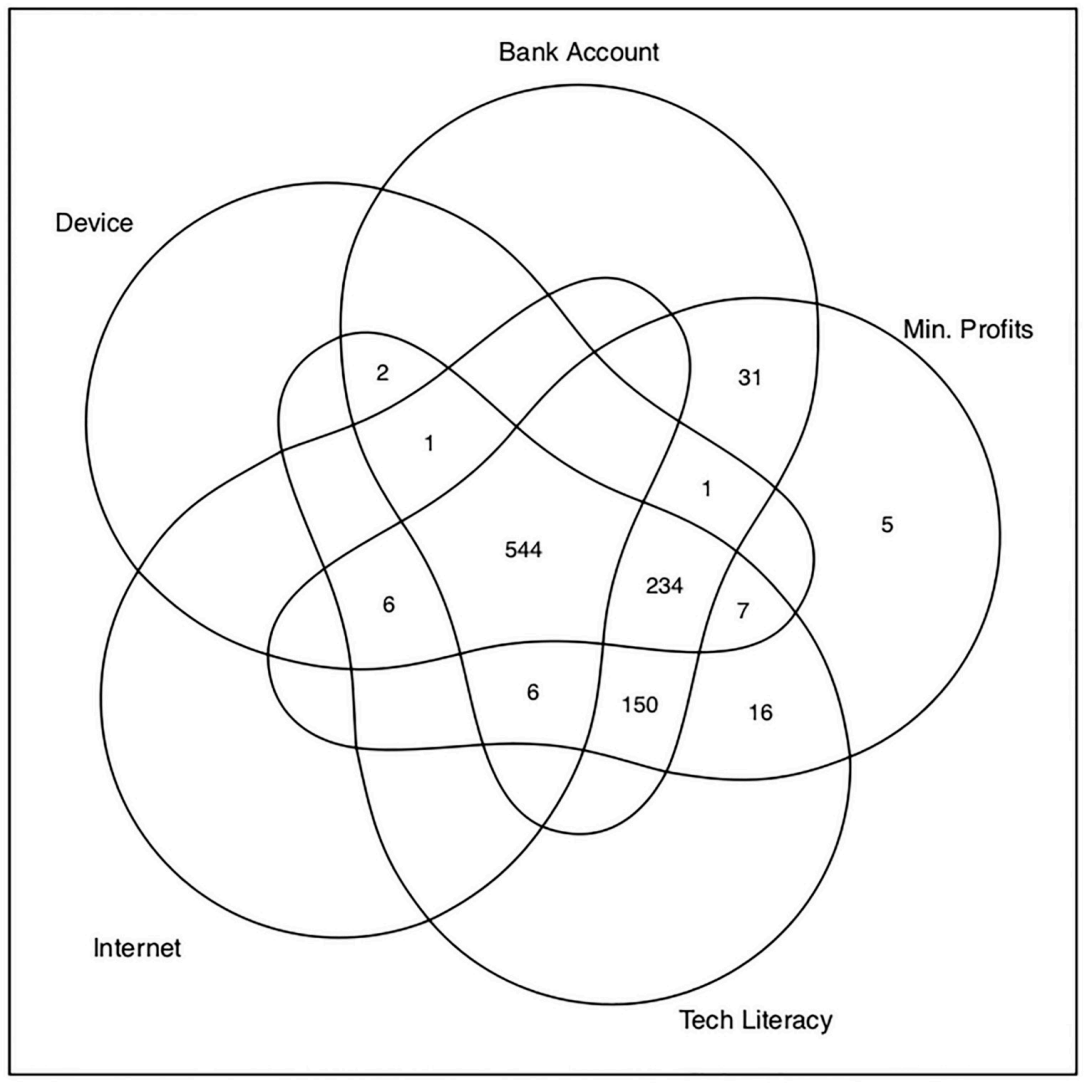
Prerequisite fulfillment by component. This Venn diagram shows the number of sample businesses (out of 1,003 total) with each possible fulfillment combination of the five prerequisites that must be satisfied to use digital payments.

In the “Already have” column of Table 4, we see that not only could most merchants satisfy the prerequisites for digital payment adoption if they so chose, but 54.24% of sample merchants actually do. Having all of the infrastructure is an endogenous choice among merchants that could predispose them to be able to use digital payments. But dividing the percentage of adopters in Table 3 by the percentage of merchants that already satisfy all the requirements in the “Already Have” column of Table 4 reveals that even among merchants satisfying all of the requirements, at most 77% of them use digital payments. In reality, the percentage of individuals with all of the requirements that are currently using digital payments is a bit lower at 67.5%, since 54 merchants (5.38% of the overall sample) report using digital payments despite not having all the requisite technology. Of these 54 merchants, notably about 91% report not having internet access. Yet it is possible that they *do* have internet access through smart phone data and misunderstood the question ascertaining whether the merchant has internet access (as it is possible that internet access was thought by some merchants to only signify WiFi or broadband connections and not cellular data). More generally, because of this confusion, there may be reason to believe that the overall number of merchants that actually do have internet access at their enterprise is higher than what is stated. This is particularly interesting given that, as we can see in Fig 1, the “binding constraint” to merchants not satisfying all five requirements seems to be internet in many cases (for over 23% of sample merchants overall). We also might worry that people may not choose to have internet access because the internet is slow or does not work very well. However, in general internet speeds in India rank 88th out of 200 countries in the world, and there is no indication from our survey data that merchants think the internet is slow. [31]

### Usage among adopters

**Fig 2:**
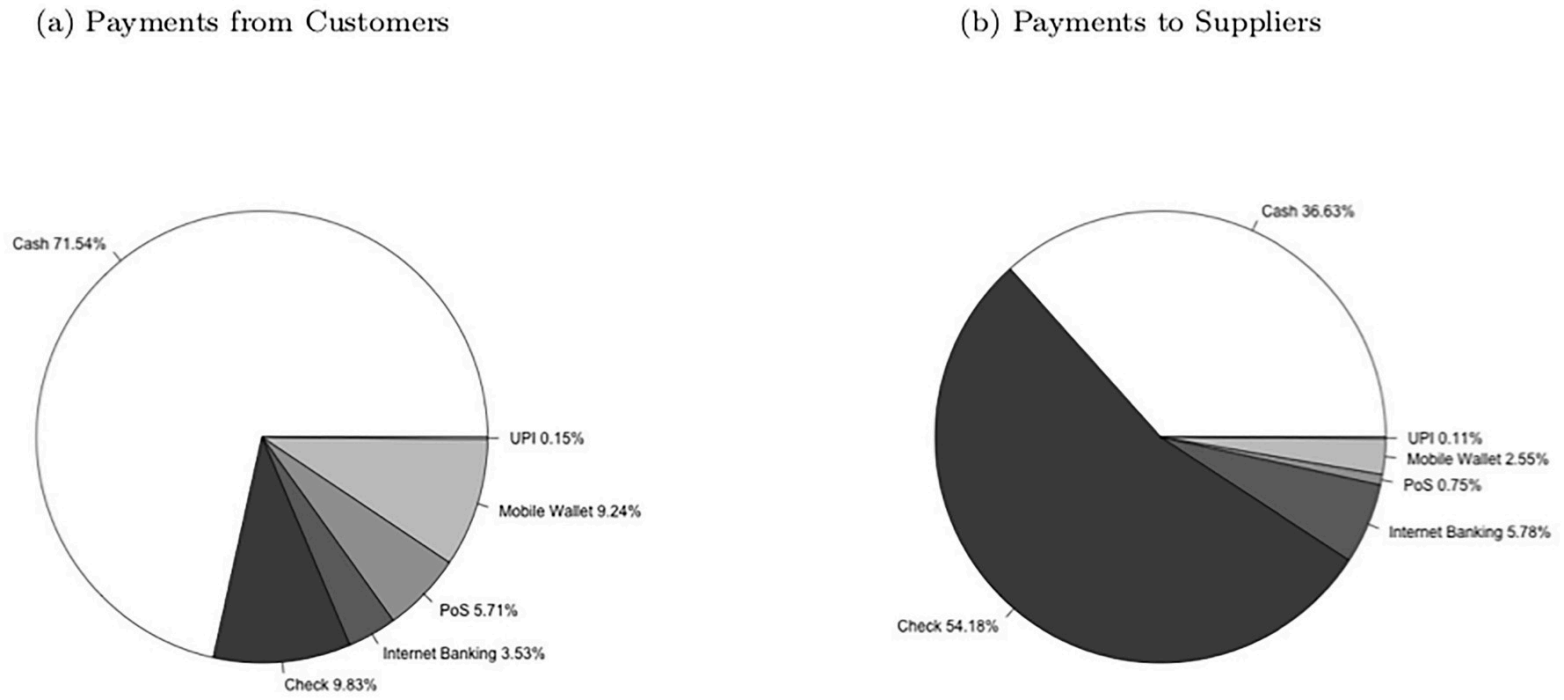
Breakdown of overall transaction values by payment method with customers and suppliers. These pie charts display the average breakdown of overall transaction values by payment method among the 421 merchants using digital payments.

Finally, we consider usage among merchants that have adopted digital payments of any type. If infrastructure and cost concerns were the main constraint restricting the adoption of digital payments and that the technology is fully optimal to use otherwise, one might expect frequent usage of digital payments among users. Our survey asked merchants what percentage of overall value they transacted through each payment medium, including cash, checks, internet banking, PoS, mobile wallets and UPI, with customers as well as with suppliers. The average breakdown of transaction values with customers and suppliers among digital payment users can be seen in Fig 2. With customers, even among digital payment using merchants, cash and checks on average make up 81.37% of transaction values, with only 18.63% coming from digital payments of any type. About half of the value paid through digital payments comes from mobile wallet transactions. Regarding payments to suppliers, similarly 90.81% of overall transaction value on average is made through cash or check. Only the remaining 9.19% of transactions are made through digital payments. Among those, over half are made through internet banking.

One might be concerned that averaging transaction value percentages over all digital payment users obscures different behaviors of users of each type of digital payments (e.g. if users of mobile wallets do most of their transactions digitally and internet banking users do almost none of their transactions digitally, averaging over both of their behaviors may not make this fact apparent). Hence we do a further breakdown of payment acceptance among users of different digital payment technologies. The results can be seen in S2 Fig. Internet banking users do 26.16% of sales in digital payments, PoS users 29.11%, mobile wallet users do 17.56%, and UPI users 25.8%. Some of the differences in this sample can generally be explained by looking at Table 3 above; users of internet banking, PoS, and UPI generally also use mobile wallets (this is necessitated by the fact that 82% of digital payment-using fixed stores use mobile wallets), hence users of internet banking, PoS, and UPI are more likely to accept multiple types of digital payments, and therefore perhaps make more overall sales using digital payment technologies. Merchants also report they prefer using internet banking and checks for purchases of larger values, and so it is sensible that relatively large portions of sales among internet banking users are made up of internet banking (and checks). As with the value of customer sales payment method breakdown, we also break down the sample further and distinguish between payment methods used to pay suppliers among users of different types of digital payment technologies in S3 Fig. Both internet banking users and UPI users transact a small but significant amount of their value paid to suppliers digitally. For internet banking users, almost a quarter of their transaction values are paid digitally, with over 80% of that quarter being comprised of internet banking payments. For UPI users, almost a quarter of their transaction values are paid digitally as well, with about half of that quarter being comprised of internet banking payments. Among users of PoS devices and among users of mobile wallets, the percentages of value to suppliers paid digitally are significantly less: at 9.1% and 7.3% respectively.

Hence even among users of digital payments, only a relatively small fraction of transactions with suppliers and customers are digital. Even among the population of merchants satisfying all of the prerequisites for digital payment adoption that have adopted, there is substantial potential for greater usage. This also provides evidence that other factors besides supply-side barriers are driving digital payment use-related decisions.

## Discussion

We have provided evidence that supply-side factors do not seem to account for low rates of digital payment adoption among sample merchants. Instead, since costs are low, the perceived benefits of adoption must also be low. In this section, we identify possible reasons that merchants may perceive benefits to be low. First, we consider merchants’ stated reasons for adopting or not adopting digital payments. Then we present evidence through regression analysis that heterogeneous beliefs about customers’ demands for digital payments and concerns about increased tax liabilities may help explain merchants’ adoption decisions.

### Stated reasons for adoption/non-adoption

Merchants were asked to provide up to three reasons underlying their digital payment adoption decision, with adopters being asked to provide reasons why they adopted, and non-adopters reasons why they had not yet adopted. The top three most cited reasons by each group of merchants are listed in Table 5. Additionally, the most common “first” reason underlying adoption decisions is starred in each category.

**Table 5:** Stated reasons for adoption and non-adoption of digital payments.

Top reasons for adopting (421 Users)	Top reasons for not Adopting (582 Non-Users)
Demonetization* (73.87%)	Lack of customer demand (54.47%)
Customer demand (62.94%)	Lack of awareness* (41.75%)
Ease of use (37.29%)	Fear of being cheated (41.75%)

Note: Merchants who use digital payments were asked to give up to three reasons why they adopted digital payments and those who do not use digital payments were asked why they did not adopt. The top three most common answers from each sample are displayed in the table, with sample sizes at the top of the row. Percent of applicable merchants who mentioned a reason are listed in parentheses below each response. An asterisk implies a response was the most common first reason reported among sample merchants (as they were able to report up to three reasons). Not all merchants reported 3 reasons; some only reported one or two reasons. Some of the wordings for reasons listed in this table are slightly modified from the original (English version) survey materials’ wording for clarity.

Among adopting merchants, the November 2016 demonetization is the most commonly reason given for adoption, with 73.9% of adopters citing it, and it being the most common reason cited first. But “demonetization” itself was a *demand-side* reason for adoption; it had no direct effect on the costs facing would-be adopters, but a possible direct benefit related to customers’ subsequent behavior. So these underlying reasons that digital payments are beneficial may lead merchants to adopt digital payments even many months after the demonetization. We see these potential benefits with some of the other commonly cited reasons: customer demand and ease of use.

Perhaps more importantly, we look at the reasons why merchants report they do *not* adopt digital payments. The most commonly cited reason is lack of customer demand for this payments technology. This is notable, as customer demand was also a top reason cited by adopters of digital payments. This suggests that adopters may have (or at least believe they have) more customers who demand to pay digitally than do non-adopters. Lack of awareness is the most common first reason cited for non-adoption, and fear of being cheated is also mentioned by a substantial fraction of merchants. These two issues could potentially be overcome with more information about digital payments and their safety being provided to merchants. If merchants are currently unaware or misinformed about using digital payments, this means that for these merchants that the perceived cost of becoming aware of these technologies and learning how to use them outweighs their perception of the benefits. Hence inducing adoption among these merchants is a matter of reducing costs, increasing benefits, or changing perceptions.

A relevant concern is that merchants’ lack of awareness could be driven by a lack of readily available information about digital payments; for example, merchants may not be aware that these methods exist. However, in our context, this is very unlikely. At the time of the survey, the Government of India was making significant public efforts to promote switching to digital payments. Information about digital payments appears to be quite abundant; sources of information about digital payments reported by surveyed merchants include: the newspaper, television, their bank, outdoor ads, social media, family and friends, other merchants, and digital payment sales people. It also is a common practice in markets to prominently post stickers with the logos of all payment types a merchant accepts (clearly providing other merchants signals of who they might be able to ask about digital adoption). Therefore, if merchants lack information necessary to adopt digital payments, it is likely that they don’t perceive the benefits to obtaining this information to be great enough.

### Possible sources of heterogeneity explaining adoption decisions

Given the stated reasons for adoption as a starting point, we try to pinpoint key sources of heterogeneity in perceived costs and benefits of digital payments that may explain adoption. First, since many adopters and non-adopters alike stated customer demand as an important factor, we consider more closely whether businesses tend to adopt digital payments based on their beliefs about how many customers demand to pay digitally. Second, given that many individuals stated that they chose not to adopt due to lack of awareness in a context where individuals are quite educated and information about digital payments is abundant, we attempt to consider reasons for non-adoption that merchants may not be willing to tell a survey enumerator. Given that adopting digital payments increases business transparency, we hypothesize and explore the idea that some merchants may not adopt because they are concerned it will increase their tax liability.

#### Heterogeneity in perceived customer demand to pay digitally

As suggested in Table 5, perceptions of customer demand to pay digitally may play a lead role in merchants’ decisions to adopt digital payments. Surveyed merchants were asked what percentage of their customers wanted to pay digitally before the November 2016 demonetization, directly following the November 2016 demonetization, and after the implementation of the GST, which referred to the current period when the survey was being implemented. Merchants tend to report an increase in the percentage of customers demanding to pay digitally after demonetization followed by a decrease in demand in the subsequent period (though not all the way to pre-demonetization levels). This certainly helps us to understand why many merchants decided to adopt “due” to the demonetization; there seem to have been large associated increases in customer demand. Additionally, if we compare merchants that have and have not adopted digital payments, in all periods, digital payments users report greater demand to pay digitally from customers on average; before demonetization 6.65% of adopters’ customers and 2.88% of non-adopters’ customers demanded to pay digitally, directly after demonetization these percentages were 26.09% and 12.36%, and at the time of the survey they were 15.10% and 5.22%. (All of these differences are statistically significant at the 0.01 level). This may be because these businesses have different clienteles with different preferences regarding paying digitally. However, we should interpret these differences cautiously, as they could simply reflect confirmation bias/ex-post decision rationalization by merchants who adopted digital payments, or better signals about the number of consumers that demand to pay digitally after adopting digital payments.

#### Heterogeneity in perceived tax liability

Digital payments promote business transparency, as they aid in creating an official transaction record for enterprises that are verified by a third party. This increased transparency is likely beneficial to merchants who want to pay their taxes correctly, as this would reduce the hassle costs associated with filing their taxes. Yet businesses that evade at least some of their taxes may worry that the increased transparency might oblige them to pay (more) taxes. Hence given heterogeneity in tax payment activity, there may be heterogeneous benefits of adopting digital payments.

Relevant to understanding the tax landscape at the time of the survey is the Goods and Services Tax (GST). The GST was passed into law on July 1, 2017, shortly before these survey exercises began. The law is essentially a reform on indirect taxes throughout India; one of the major relevant changes is that various players along the supply chain for a good or service can get tax refunds such that they have only paid tax on the value added on their step in the supply chain. Because in order to get refunded properly, the merchant must have detailed records of purchased items and taxes already paid for them, GST especially incentivizes better record-keeping. In Rajasthan, in most cases, businesses with a taxable supply turnover of over Rs. 2,000,000 were required by the reform to register for GST. According to self-reports, about 53% of sample businesses are mandated to pay GST.

We can identify which enterprises likely pay (at least some) taxes by looking at which have a tax identification number and/or have registered for GST. At the time of the survey (shortly after GST was mandated) businesses that adopted digital payments were significantly more likely to have a valid tax identification number (74.1% of individuals who adopted digital payments versus 48.1% of merchants who had not yet adopted). This is likely partially due to fewer businesses in the non-adopter sample being legally required to pay taxes, as they are statistically significantly smaller businesses on average, in terms of sales, profits, and number of employees. Yet, even conditional on businesses reporting that they are mandated to register for GST, digital payment users are significantly more likely to report that they are already registered to pay GST (78.4% of GST mandated payment adopters versus 60.6% of GST mandated payment non-adopters). The discrepancy in the percentages of digital payment adopters and non-adopters that were already paying taxes at the time of the survey reinforces the distinction hypothesized above: for people already mostly paying taxes, digital payments likely make this process easier, but for those not already paying taxes, the increased transparency of digital payments may be seen as a negative. We also see some evidence that digital payment users think digital payments will make GST compliance easier in that 38.48% of digital payment adopters think GST will cause their suppliers to switch over to digital payments, while only 19.42% of non-adopters think this is the case. So heterogeneity in beliefs about how GST and tax payment in general interacts with digital payments may also help to explain digital payment adoption decisions.

#### Predicting digital payment adoption with customer demand and tax behavior

While these comparisons between digital payment adopters and non-adopters are informative, we also know that there are other important differences between these firms, both observable and unobservable. Some of these differences may simultaneously affect customer demand for digital payments, tax payment behavior, and digital payment adoption. To address this issue, we estimate a linear probability model (using ordinary least squares) to see how tax paying behavior and customer demand beliefs predict which firms adopt digital payments, controlling for other observable characteristics. (S2 Appendix contains tables showing a t-test of means of all dependent variables used in the regression between adopters and non-adopters). These results can be found in Table 6.

**Table 6:** Predicting digital payments adoption with tax registration status and reported customer demand.

	Dependent variable: Digital payments adoption indicator				
	(1)	(2)	(3)	(4)	(5)
Have tax ID no.	0.140*** (0.036)	0.128*** (0.036)	0.090** (0.035)	0.064* (0.037)	0.069* (0.035)
GST registered	0.105*** (0.039)	0.114*** (0.040)	0.053 (0.044)	0.056 (0.046)	0.064 (0.044)
Customer demand (Pre Nov. 2016)	−0.445** (0.202)	−0.495** (0.201)	−0.641*** (0.180)	0.288* (0.168)	−0.616*** (0.180)
Customer demand (Post Nov. 2016)	0.734*** (0.102)	0.718*** (0.100)	0.288*** (0.096)		0.293*** (0.095)
Customer demand (Now)	0.926*** (0.158)	0.845*** (0.154)	0.917*** (0.143)		0.884*** (0.142)
Industry FE	N	Y	Y	Y	Y
Enterprise controls	N	N	Y	Y	Y
Business owner controls	N	N	N	Y	Y
Observations	1,003	1,003	1,003	1,003	1,003
R^2^	0.239	0.280	0.443	0.309	0.453
Adjusted R^2^	0.235	0.253	0.411	0.275	0.416
F Statistic	105.38***	10.42***	13.71***	10.96***	12.32***

Note:

* *p* < 0.1;

** *p* < 0.05;

*** *p* < 0.01

Standard errors are robust to heteroskedasticity. Enterprise controls include: an indicator for whether the business is registered, business age (under current ownership), business age squared, number of employees, sales, profits, whether the business has a bank account, whether the business has an internet-accessing device, whether the business has internet access, whether the business has technological literacy (owner or one of the employees as defined in previous sections), percentages of transactions that are business-to-business (or b2b), whether the business is mandated to pay GST (self-reported), number of customers who visit each week, the share of repeat customers, number of suppliers who the business transacts with each week, an indicator of whether the business offers delivery service, an indicator for whether the business sells goods online, an indicator for whether the business provides credit to customers, weekly cash inflows, an indicator for whether the business has an outstanding loan, and indicators for whether the business is a convenience store, specialty store, wholesaler retailer, and service provider. Business owner characteristics include: whether they have an Aadhaar card, whether they have a PAN card, a gender indicator, a (Hindu) religion indicator, a social class indicator (whether they are from an scheduled caste/tribe or other backwards class), an indicator for whether they have a 10th grade education or above, an indicator for whether someone in their household has a 10th grade education or above, household size, and household income.

The dependent variable is a binary indicator of whether or not a merchant had adopted any of the four types of digital payment technologies at the time of the survey. The coefficient estimates can be interpreted as reflecting variation in the probability of adoption. There are 5 explanatory variables of interest. “Have tax ID no.” is an indicator of whether the merchant had a TIN, TAN, or GST number at the time of the survey, “GST registered” is an indicator of whether the merchant was registered for GST at the time of the survey, “Customer demand (Pre Nov. 2016)” denotes merchants’ self-reported proportion (between zero and one) of customers demanding to pay digitally before the November 2016 demonetization, “Customer demand (Post Nov. 2016)” denotes the same proportion for directly following the demonetization, and “Customer demand (Now)” denotes the same proportion at the time of the survey in August 17th. The caveats associated with especially the customer demand variables from previous sections certainly still apply.

Columns 2–5 have fixed effects for industry category. Columns 3–5 have controls for enterprise characteristics, and Columns 4–5 also control for business owner characteristics. Column 4 differs from Column 5 by only including customer demand pre-demonetization. Table 5 suggests that many merchants adopted digital payments as a consequence of demonetization. If adopting digital payments is associated with ex-ante rationalization or learning about demand for digital payments, we would expect customer demand estimates for digital payments may be biased upwards for all periods following the demonetization for digital payment users. We therefore expect customer demand before demonetization to be less susceptible to such bias. We therefore restrict Column 4 to exclude the potentially biased demand indicators post-demonetization.

Having a tax ID number (suggesting the merchant pays at least some taxes) implies a 6% higher probability of having adopted digital payments. Meanwhile, having registered for GST is not significantly related to digital payment adoption after accounting for enterprise characteristics. Beliefs about customer digital payment demand are correlated significantly with digital payment adoption. Using only pre-demonetization demand, a 10 percentage point increase in percentage of customers demanding digital payments implies an approximately 2.9% increase in the probability of adopting digital payments (Column 4). When including demand for digital payments in the two periods following demonetization are included, the effect of customer demand in the pre-demonetization period becomes negative, while it is positive in the other two post periods. This is possibly because customer demand in the pre-period is positively correlated with demand in both post periods (correlations are 0.53 and 0.6 with demand post-demonetization and at the time of the survey respectively). But when including all three periods, a 10 percentage point increase in customer demand for digital payments in the current period is associated with an increase in the likelihood of adoption by 9.2%. Hence, we see that even when controlling for various other business and personal observable characteristics, tax payment and perceived customer demand to pay digitally significantly correlate with merchant adoption decisions.

## Conclusion

In this paper, we explored whether the lack of digital payment adoption observe in India, and specifically among small-scale fixed store merchants in Jaipur, is due to supply-side costs of adoption: specifically, obtaining a bank account, an appropriate device, internet access, profits to cover usage fees, and technological literacy. Identifying the number of merchants who could feasibly satisfy all of these prerequisites (over 98%) and how many merchants already satisfy these prerequisites (over 50%) reveals lack of support for the hypothesis that these requirements are binding constraints to digital payment adoption (currently only around 40%) in this population. Instead, we conclude that heterogeneity in perceived benefits of adoption may play a more central role in adoption decisions. We propose some possible sources of heterogeneity, showing that digital payment adopters believe more of their customers demand to pay digitally and are more likely to be tax payers. Additionally, we show that controlling for other observable characteristics of businesses and merchants, perceived customer demand to pay digitally and tax payment behavior are both highly predictive of digital payment adoption.

We conclude that if policymakers in India wish to encourage additional adoption of digital payments by small-scale merchants, subsidizing adoption is unlikely to be effective among small-scale fixed store merchants. Customer demand, however, may be an important part of the decision. Therefore, India’s recent policy of incentivizing *both* customers and merchants to adopt digital payments (specifically UPI) could provide a push toward a more digital economy by all parties, much in the way the demonetization did (at least in the short run). Our investigation suggests that experimental tests of joint incentives for customers and merchants could be a fruitful area for future research. Additionally, understanding which merchants will not further formalize by adopting digital payments in order to avoid taxes will be critical in forming policies regarding the intersection of digital payments and taxes. Whether these results explain adoption patterns for other types of merchants or businesses in India, and whether adoption patterns differ later in the technological diffusion process (one year after the survey was done, there was already a roughly 19-fold increase [32] in UPI usage, and a 5% increase in electronic payment volumes generally [33]), are additional areas of future research.

## Supporting information

S1 Fig Distribution of annual profits (as compared to annual digital payments-related costs. Empirical cumulative density function of annual profits (figure truncated at profits of INR 2,500,000 for visual clarity).
(TIFF)

S2 Fig Breakdown of customer payments received by technology user type. Pie charts of percentages of value received from customers by each payment method stratified by digital user type, for the four types of digital payment technology.
(TIFF)

S3 Fig Breakdown of supplier payments made by technology user type. Pie charts of percentages of value paid to suppliers with each payment method stratified by digital user type, for the four types of digital payment technology.
(TIFF)

S1 Appendix Sampling procedure. This document contains a detailed description of how businesses were identified in the listing exercise and how they were then selected to create our survey analysis sample.
(PDF)

S2 Appendix Tests of means of observable characteristics for adopters vs. non-adopters. This document contains tables that perform simple t-tests of means comparing all explanatory variables used in the regression analysis among the digital payment adopter and digital payment non-adopter samples.
(PDF)
