# Supplementary figures and images for: What explains low adoption of digital payment technologies? Evidence from small-scale merchants in Jaipur, India
Source: PLoS One. 2019 Jul 31;14(7):e0219450. doi: 10.1371/journal.pone.0219450 (PMC6668901; doi:10.1371/journal.pone.0219450)

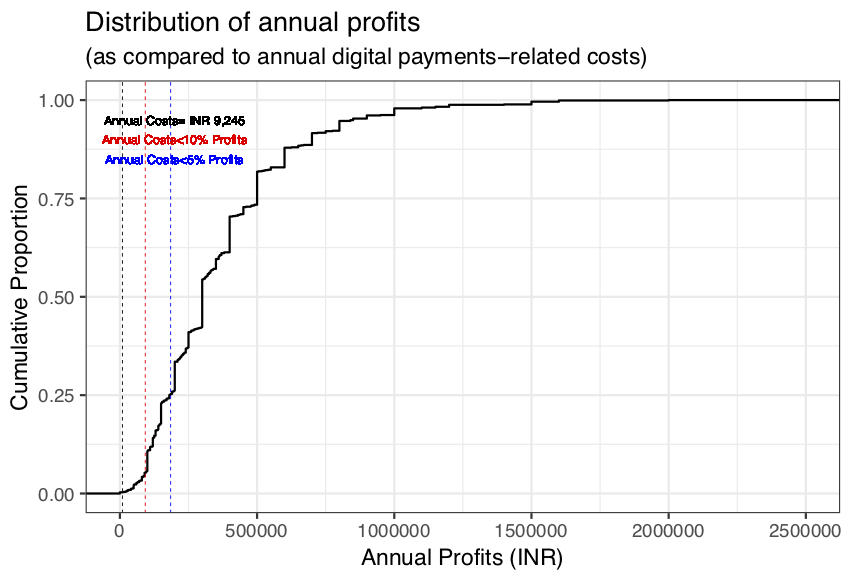

Supplement: S1 Fig — Empirical cumulative density function of annual profits (figure truncated at profits of INR 2,500,000 for visual clarity). (TIFF) [file pone.0219450.s001.tiff]

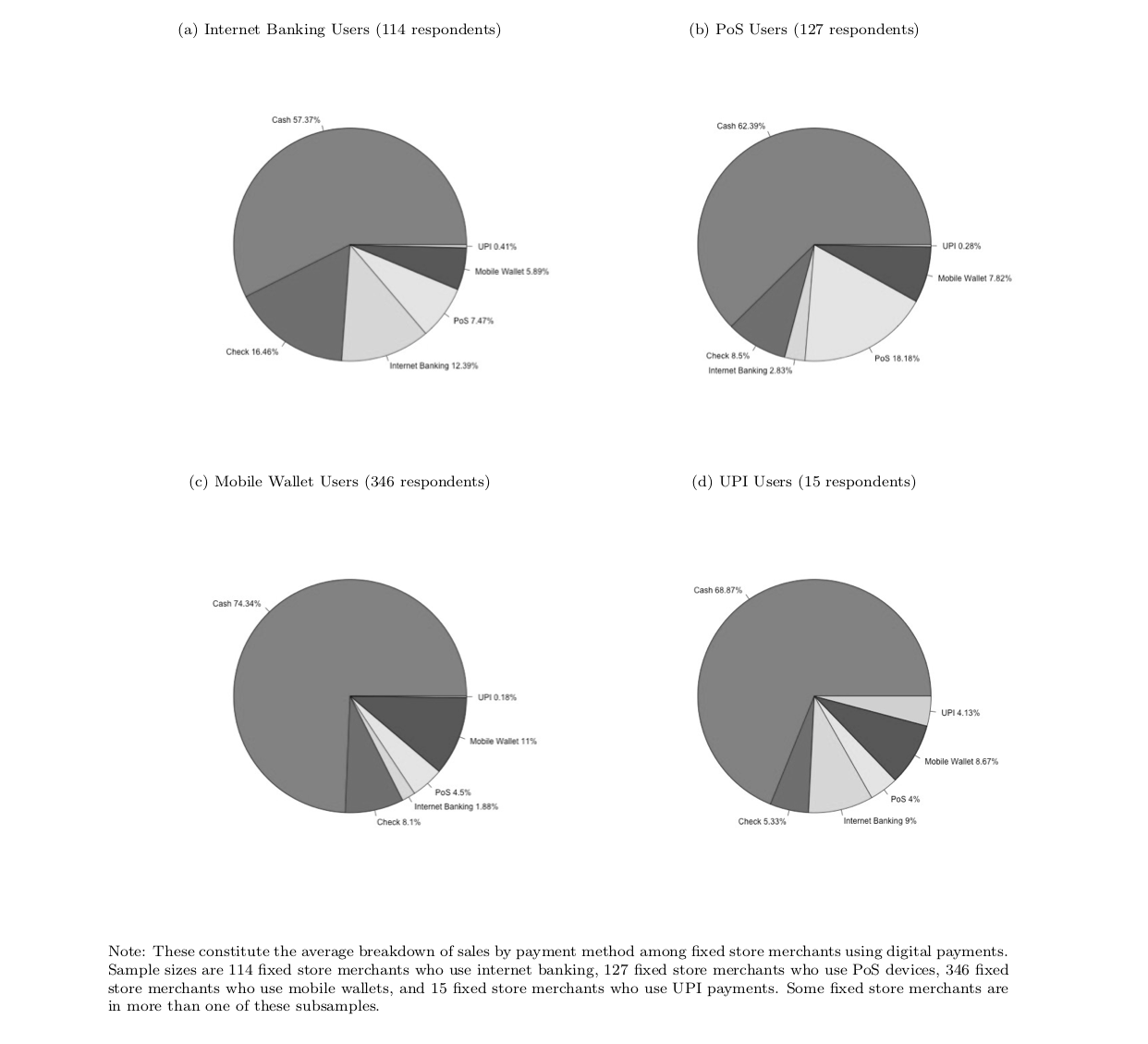

Supplement: S2 Fig — Pie charts of percentages of value received from customers by each payment method stratified by digital user type, for the four types of digital payment technology. (TIFF) [file pone.0219450.s002.tiff]

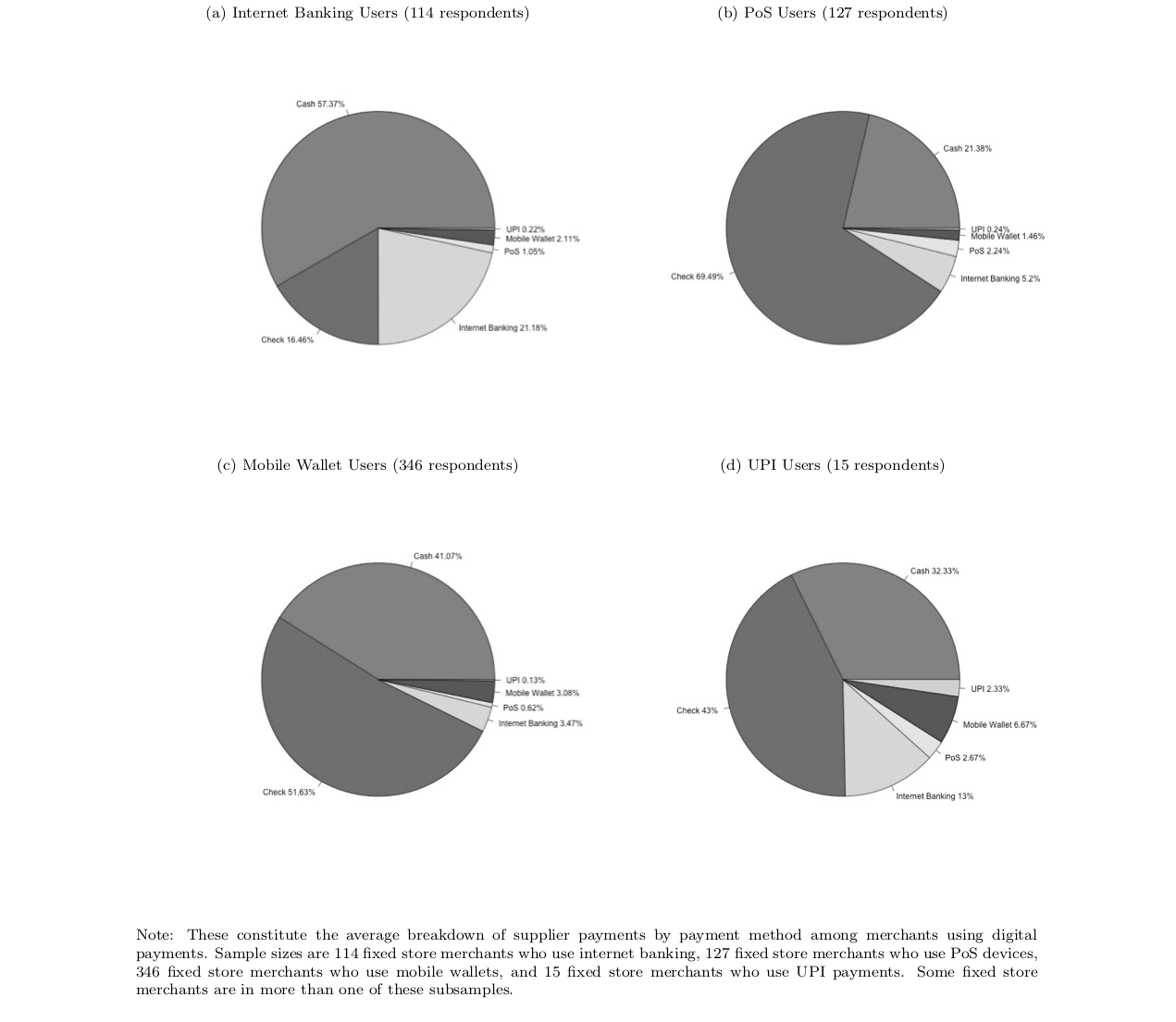

Supplement: S3 Fig — Pie charts of percentages of value paid to suppliers with each payment method stratified by digital user type, for the four types of digital payment technology. (TIFF) [file pone.0219450.s003.tiff]
