# Supplementary material for: What explains low adoption of digital payment technologies? Evidence from small-scale merchants in Jaipur, India
Source: PLoS One. 2019 Jul 31;14(7):e0219450. doi: 10.1371/journal.pone.0219450 (PMC6668901; doi:10.1371/journal.pone.0219450)
Supplement: S1 Appendix — This document contains a detailed description of how businesses were identified in the listing exercise and how they were then selected to create our survey analysis sample. (PDF) [file pone.0219450.s004.pdf]

S1 Appendix: Sampling procedure

Sampling happened in two stages, a listing or census, and a main sample. First, thirty market areas within Jaipur were identified and mapped. In each market, field staff were given maps of the market locations, started from the entrance of the market, and visited shops in a clockwise direction until they had completed a listing of 200 fixed stores. For sampling purposes, a “fixed store” was defined as an enterprise where the activity carried out is within fixed premises or in a permanent structure outside the household. Additionally, each physical store itself was treated as a sole unit; if an enterprise had multiple branch locations, each was treated as a separate fixed store for the purpose of the listing. Surveyors would ask a set of listing questions at each fixed store of the owner of the business.

The listing stage resulted in data on 6,011 fixed stores. Using this as the population of interest, a stratified random sampling strategy was used in order to select the final sample of 1,003 fixed stores in the main survey. First, given the major industry categories present in the sample (see Table 1), the industries in the census were broken into 5 groups: General Store, Apparels, fabrics, and furnishings, Food Service Activities, Manufacturing, Toiletries, cosmetics, and gift shops, and Other.

Table 1. Fixed store census data top industries.

Industry Type	Number of Businesses	% Businesses
General store	1294	21.53%
Apparels, fabrics and furnishing	1177	19.58%
Food service activities	612	10.18%
Manufacturing (including repair and installation of machines)	467	7.77%
Toiletry, cosmetics and gifts shops	450	7.49%
Other	2011	33.46%

Then every business was given a ”digital readiness score” based on their annual turnover, internet access, technology use, bank account usage, loan behavior, and digital payment usage. The calculation of this score involves summing up scores from the 8 categories in Table 2. Businesses from each industry group were separated into quartiles based on their digital readiness score. The corresponding industry by digital readiness quartile breakdown can be seen in Table 3).

Table 2. Digital readiness score calculation.

Category	Score Chart
Annual Turnover	Based on Percentile
Access to Internet	Yes=8, No=0
Usage of Computer	Yes=2, No=0
Usage of Laptop	Yes=2, No=0
Usage of Landline	Yes=2, No=0
Usage of Mobile	Yes=2, No=0
Type of Bank Account	N/A=0, Savings=4, Current=6, Both=8
Usage of digital payment modes	N/A=0, Any one=2, Any two=4, Any three=6, All four=8

The sample was stratified by industry category and by “digital readiness” quartile. Within an industry category each quartile was sampled equally, save for the “Other”

Table 3. Fixed store census data frequencies by industry category and quartile of digital readiness score.

Inudstry Category	1st Quartile	2nd Quartile	3rd Quartile	4th Quartile	Total
General store	387	359	335	213	1294
Apparels, fabrics and furnishing	189	284	331	373	1177
Food service activities	269	146	142	55	612
Manufacturing	179	86	105	97	467
Toiletry, cosmetics and gifts shops	142	126	114	68	450
Other	572	455	546	438	2011
Total Stratified Sample					6011

Table 4. Main sample data top industries.

Industry Category	# Businesses	% Sample	% Listing
General Store	124	12.4	21.5%
Apparels, fabrics and furnishing	186	18.5	19.6
Food service activities	120	12.0	10.2
Manufacturing (including repair and installation of machines)	89	8.9	8.0
Toiletry, cosmetics and gifts shops	86	8.6	7.5
Other	398	39.7	33.5

category, which had quartiles sampled in the ratio 25:15:12:11. By comparing the final two columns of Table 4 one could construct sampling weights; e.g., general stores are undersampled relative to their proportion in the listing, so by reweighting these observations by the ratio 21.5/12.4 one could obtain sample statistics that matched statistics computed for the listing. Note, however, that we have chosen *not* to do this in the main analysis of the paper, preferring to avoid the additional complexity involved. We do not believe our main conclusions would be affected by such a re-weighting.
