# Supplementary material for: What explains low adoption of digital payment technologies? Evidence from small-scale merchants in Jaipur, India
Source: PLoS One. 2019 Jul 31;14(7):e0219450. doi: 10.1371/journal.pone.0219450 (PMC6668901; doi:10.1371/journal.pone.0219450)
Supplement: S2 Appendix — This document contains tables that perform simple t-tests of means comparing all explanatory variables used in the regression analysis among the digital payment adopter and digital payment non-adopter samples. (PDF) [file pone.0219450.s005.pdf]

S2 Appendix: Tests of means of observable characteristics for adopters vs. non-adopters

Each table below presents a Welch two sample t-test of means comparing each indicated variable for businesses (and corresponding business owners) who have reported adopting any form of digital payments and for those who have not. Means are listed with standard errors in parenthesis below. The p-value corresponds to the t-test performed in each row. * denotes $P < 0.1$, ** denotes $P < 0.05$ and *** denotes $P < 0.01$.

Table 1. Tests of means of explanatory variables of interest for adopters vs. non-adopters.

Characteristic	Adopters	Non-adopters	P-Value
Business has tax ID no.	74.1%<br>(2.14)	48.8%<br>(2.07)	0.000***
Business has registered for GST	53.4%<br>(2.43)	26.5%<br>(1.83)	0.000***
Percent of customers demanding to pay digitally (Pre Nov. 2016)	6.65%<br>(0.514)	2.88%<br>(0.277)	0.000***
Share of customers demanding to pay digitally (Post Nov. 2016)	26.1%<br>(0.936)	12.4%<br>(0.574)	0.000***
Share of customers demanding to pay digitally (Now)	15.1%<br>(0.724)	5.21%<br>(0.315)	0.000***

Table 2. Tests of means of observable business owner characteristics for adopters vs. non-adopters.

Characteristic	Adopters	Non-adopters	P-Value
Owner has an Aadhaar card	99.5%<br>(0.336)	98.8%<br>(0.452)	0.197
Owner has an PAN card	95.5%<br>(1.01)	91.2%<br>(1.17)	0.006***
Owner is male	99.0%<br>(0.473)	98.6%<br>(0.483)	0.530
Owner is Hindu	89.8%<br>(1.48)	80.6%<br>(1.64)	0.000***
Owner is a member of scheduled caste/tribe or OBC	24.0%<br>(2.08)	41.8%<br>(2.05)	0.000***
Owner has 10th grade education	88.8%<br>(1.54)	72.5%<br>(1.85)	0.000***
Someone in household has 10th grade education	97.1%<br>(0.812)	91.2%<br>(1.17)	0.000***
Household size	6.41 people<br>(0.196)	6.14 people<br>(0.147)	0.275
Annual household income	Rs. 81,591<br>(583,834)	Rs. 217,895<br>(434,091)	0.000***

Table 3. Tests of means of observable business characteristics for adopters vs. non-adopters.

Characteristic	Adopters	Non-adopters	P-Value
Business is registered	92.2%<br>(1.31)	77.5%<br>(1.73)	0.000***
Business age (Current Ownership)	16.7 years<br>(0.689)	16.4 years<br>(0.535)	0.744
Number of employees	2.43<br>(0.0902)	1.75<br>(0.0610)	0.000***
Annual sales	Rs. 2,562,340<br>(148,244)	Rs. 1,847,457<br>(96,015)	0.000***
Annual profits	Rs. 442,481<br>(25,580)	Rs. 337,287<br>(13,990)	0.000***
Business has bank account	99.3%<br>(0.410)	94.7%<br>(0.932)	0.000***
Business has internet-capable device	98.6%<br>(0.578)	65.3%<br>(1.97)	0.000***
Business has internet access	88.4%<br>(1.56)	31.8%<br>(1.93)	0.000***
Business has technological literacy	100%<br>(0.00)	81.8%<br>(1.60)	0.000***
Percentage of Transactions that are B2B	24.1%<br>(1.43)	16.6%<br>(1.15)	0.000***
Business is mandated to pay GST	68.2%<br>(2.27)	43.6%<br>(2.06)	0.000***
Number of customers visiting weekly	223 customers<br>(71.3)	153 customers<br>(11.7)	0.332
Share of repeat customers	43.3%<br>(1.19)	43.2%<br>(1.05)	0.960
Number of suppliers transacted with weekly	2.34 suppliers<br>(0.268)	2.21 suppliers<br>(0.145)	0.656
Business offers delivery service	5.94%<br>(1.15)	2.23%<br>(0.613)	0.005***
Business sells goods online	2.14%<br>(0.706)	0.172%<br>(0.172)	0.007***
Business sells goods online	2.14%<br>(0.706)	0.172%<br>(0.172)	0.007***
Business offers credit to customers	30.2%<br>(2.24)	29.0%<br>(1.88)	0.700
Weekly cash inflows	Rs. 62,611<br>(20,532)	Rs. 24,327<br>(2,168)	0.064*
Business has outstanding loan	3.56%<br>(0.904)	2.58%<br>(0.657)	0.378
Business is a "convenience" store	39.0%<br>(2.40)	44.3%<br>(2.06)	0.088*
Business is a "specialty" store	34.4%<br>(2.32)	28.7%<br>(1.88)	0.054*
Business is a wholesale store	17.1%<br>(1.84)	13.1%<br>(1.40)	0.080*
Business is a service provider	4.51%<br>(1.01)	8.08%<br>(1.13)	0.019**
